# Case report: Heterozygous variation in the IGHMBP2 gene leading to spinal muscular atrophy with respiratory distress type 1

**DOI:** 10.3389/fneur.2024.1289625

**Published:** 2024-05-30

**Authors:** Chaoai Zhou, Zefu Chen, Qiqing Chen, Xiaowei Feng

**Affiliations:** ^1^Department of Pediatrics, Hainan General Hospital (Hainan Affiliated Hospital of Hainan Medical University), Haikou, China; ^2^Department of Ultrasound, Hainan General Hospital (Hainan Affiliated Hospital of Hainan Medical University), Haikou, China

**Keywords:** SMARD1, IGHMBP2, neuromuscular genetic disease, symmetrical distal limb weakness, respiratory failure

## Abstract

A rare autosomal recessive genetic disease is spinal muscular atrophy with respiratory distress type 1 (SMARD 1; OMIM #604320), which is characterized by progressive distal limb muscle weakness, muscular atrophy, and early onset of respiratory failure. Herein, we report the case of a 4-month-old female infant with SMARD type 1 who was admitted to our hospital owing to unexplained distal limb muscle weakness and early respiratory failure. This report summarizes the characteristics of SMARD type 1 caused by heterozygous variation in the immunoglobulin mu DNA binding protein 2 (IGHMBP2) gene by analyzing its clinical manifestations, genetic variation characteristics, and related examinations, aiming to deepen clinicians’ understanding of the disease, assisting pediatricians in providing medical information to parents and improving the decision-making process involved in establishing life support.

## Introduction

A rare autosomal recessive neuromuscular genetic disease is spinal muscular atrophy with respiratory distress type 1 (SMARD1; OMIM #604320), the incidence of which is 1:100,000. The primary symptoms include progressive and symmetrical distal limb weakness, muscle atrophy, and early respiratory failure owing to diaphragmatic paralysis, which is commonly observed in infants and young children (1). This change occurs owing to mutations in immunoglobulin mu DNA binding protein 2 (IGHMBP2) (2). The most prominent clinical feature is early involvement of the respiratory system, which initially manifests as weak crying, frequent respiratory infections, and swallowing difficulties; rapid progression of the disease; and sudden respiratory distress that is often caused by diaphragmatic paralysis and respiratory failure (3). Therefore, significant challenges exist in the early diagnosis, genetic counseling, prevention, and treatment of SMARD1. Herein, we report a rare case in which SMARD1 was caused by an IGHMBP2 gene heterozygous mutation in a patient admitted to our pediatric department to increase the understanding of the disease among clinical physicians.

## Case introduction

**Figure 1:**
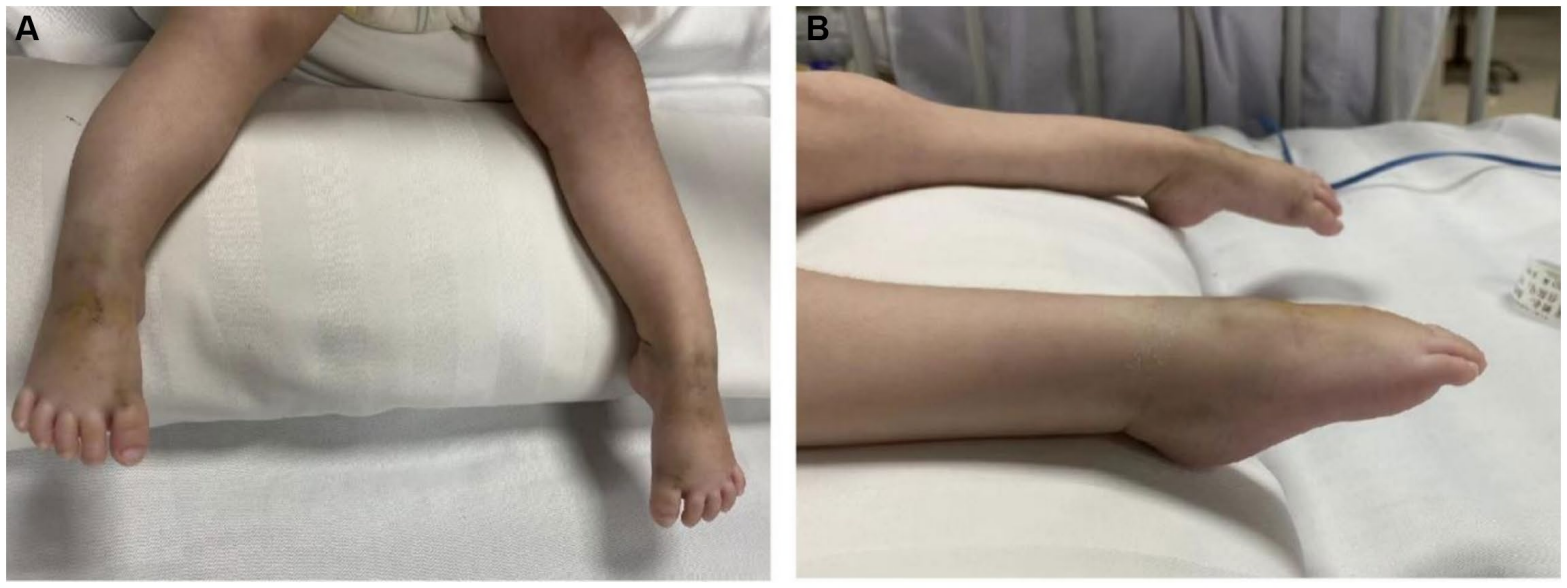
**(A,B)** Bilateral ankle joint contracture, foot droop, and difficulty in dorsiflexion in the patient.

A 4-month-old female patient reported muscle weakness for more than a month without any obvious cause. Her mother’s medical history did not reveal pregnancy-induced hypertension or diabetes, and her nutritional and health status was good. During pregnancy, her mother experienced a decrease in fetal movement; however, compared with other children, the child was born full-term, with a birth weight of 2 kg, moderate crying at birth, a lower voice, and moderate limb movement. There was no history of birth asphyxia. Initially, the patient presented with weakness in both lower limbs, which gradually worsened and affected both upper limbs. Therefore, when she was 2-month-26-day-old, she was taken to a local hospital for a double hip joint radiograph, which indicated congenital dysplasia of the left hip joint. After rehabilitation treatment was administered for 1 month at 2-month-28-day-old (for which specific information was unavailable), there was a gradual worsening of muscle weakness in the limbs, accompanied by a progressive decrease in crying. To seek further treatment, her guardians sought medical attention at our hospital, and she was admitted to the outpatient department as a case of ‘muscle weakness awaiting investigation.’ The child had poor growth and development, lagging behind children of the same age and sex. Initial examination showed that the child has limb weakness, with lower limb muscle strength of level 2. The lower limbs move in a weightless state but cannot resist gravity. The lower limbs cannot be lifted off the bed. And the upper limb muscle strength was level 3. The upper limbs can be lifted off the bed but cannot resist external resistance. The specialized physical examination demonstrated the following findings: The child’s weight was 4 kg, and mental fatigue and the average reaction were noted. The chest was concave and presented as a funnel chest, with lower and upper limb muscle strengths of levels 2 and 3, respectively. The muscle tension was weak, and the deformed spine exhibited an S-shaped bending deformity, bilateral ankle joint contractures, foot sagging, and difficulty in dorsiflexion (Figure 1). Bilateral superficial abdominal wall reflexes led out symmetrically. Examination revealed weak knee reflexes with negative Kernig, Brudzinski and Babinski signs.

## Relevant examinations for admission improvement

**Figure 2:**
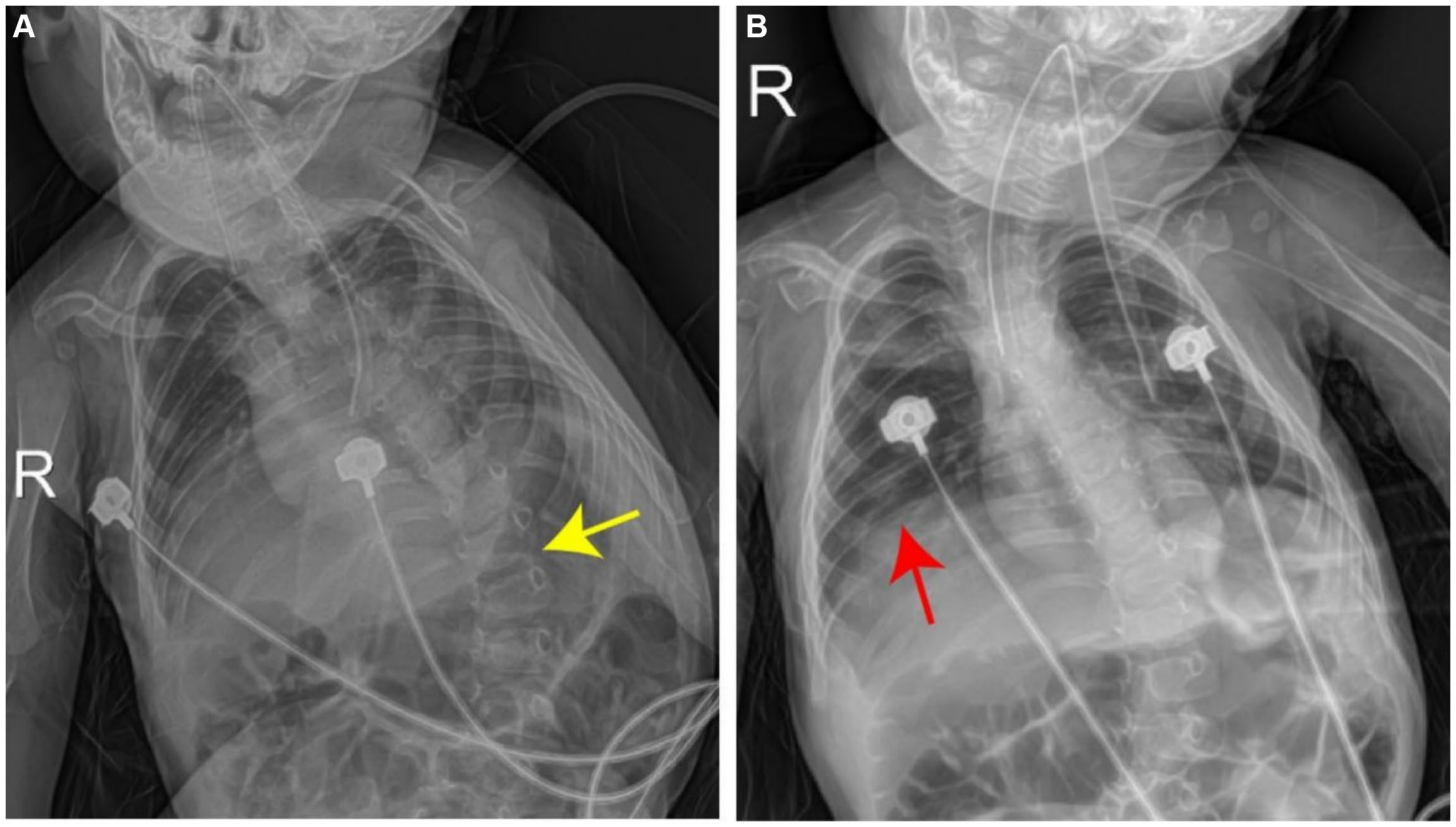
Chest radiograph shows left lateral curvature of the thoracic spine (**A**, yellow arrow), accompanied by elevation of the right diaphragm (**B**, red arrow).

Blood gas analysis revealed the following findings: pH, 7.241; PCO_2_, 59.2 mmHg; PO_2_, 63 mmHg; Be, −2 mmol/L; and SO_2_, 87%, indicating hypoxia and carbon dioxide retention. The levels of myocardial enzymes were as follows: phosphocreatine kinase (CK) slightly increased by approximately 102.2 U/L (reference value 40–100 U/L), and CK isoenzyme (CK-MB) increased by approximately 54.2 U/L (reference value 0–24 U/L). There were no significant abnormalities in troponin T levels; routine blood test results; blood ammonia levels; electrolyte levels; liver or kidney function; coagulation; C-reactive protein levels; brain natriuretic peptide levels; TORCH-IgM levels; epidermolysis bullosa; or cytomegalovirus infections. A joint ultrasound revealed a type IIa hip joint, which was recommended for follow-up and observation. The lower limb muscle strength of the child was lower than that of the other children, and under normal conditions, the left foot was in a dorsiflexion position with poor plantar flexion. A chest radiograph revealed bilateral enhanced lung markings. The deformed spine exhibited an S-shaped bending deformity with an elevated right diaphragm (Figure 2). An electroencephalogram revealed abnormal electroencephalogram (background activity: basic rhythm moderate to high amplitude 2–3 c/s delta activity and a small amount of moderate amplitude 4 c/s delta activity, with irregular manifestations). Electromyography demonstrated that the peripheral nerves examined in the limbs were generally normal when the action potential was latent, and the amplitude was significantly low or even not elicited. Occasionally, denervated potentials were observed in the limbs and trunk muscles (with obvious distal muscles), and motor unit potential morphology was generally normal with poor recruitment. Muscle weakness antibody determination revealed ryanodine receptor antibodies (RyR-Ab) (+), Titin antibodies (Titin-Ab) (+), acetylcholine receptor antibodies (AChR-Ab) (−), and muscle-specific kinase antibodies (MuSK-Ab) (−). Detection of spinal muscular atrophy (SMA) showed no pathogenic copy number variation in survival motor neuron 1 (SMN1) and survival motor neuron 2 (SMN2) genes.

### Gene testing

**Figure 3:**
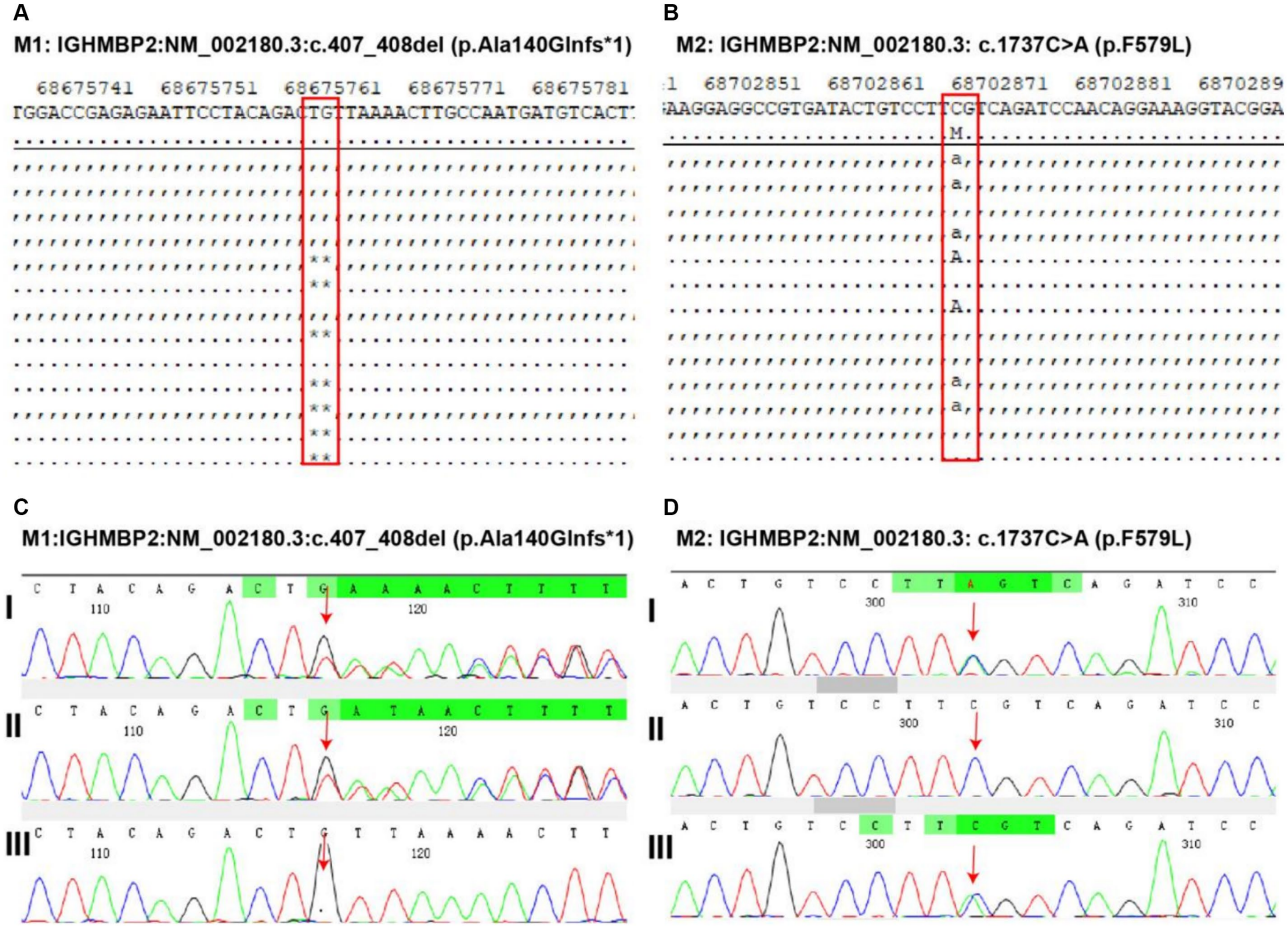
Discovery and validation of gene mutations related to pediatric patient. Two heterozygous variants in the immunoglobulin mu DNA binding protein 2 (IGHMBP2) gene of the patient. M1: IGHMBP2:NM_002180.3:c.407_408del (p.Ala140Glnfs*1) **(A)** and M2: IGHMBP2: NM_002180.3: c.1737C > A (p.F579L) **(B)** mutation discovery. The validation of M1 (**C**, I: heterozygous variation in the child, II: heterozygous variation in the father of the child, III: heterozygous variation not found in the mother of the child) and M2 (**D**, I: heterozygous variation in the child, II: heterozygous variation not detected in the father of the child, III: heterozygous variation in the mother of the child) variants indicates that the first-generation sequencing results show that these two variants are true and reliable, and are, respectively, inherited from the parents, forming a composite heterozygous variation.

Whole-exome sequencing was performed at Shanghai We-Health Biomedical Technology Co., Ltd. Genomic DNA was extracted from peripheral leukocytes from the patient using a commercial kit (TIANGEN, China). The quantity/quality of the DNA was assessed using an Onedrop OD1000 spectrophotometer and agarose gel electrophoresis. Exome capture was performed with a Twist custom panel (WES), and 150-base pair paired-end sequencing was executed using the Illumina HiSeq4000 platform (San Diego, CA). The raw reads were aligned by a sequencing company using the Burrows–Wheeler Aligner (BWA) and SAMtools. After removing duplicates from the sorted alignment using Picard, germline variants were identified via the GATK (4.2.0.0) pipeline, and somatic variants were identified via the VarScan (v2.4.3) pipeline. The spinal muscular atrophy-1-related gene SMN1 was analyzed first. Moreover, genes related to other muscle disorders were also analyzed. The results showed that two heterozygous variants in the IGHMBP2 gene of the patient, M1: NM_002180.3:c.407_408del (p.Ala140Glnfs*1) and M2: NM_002180.3:c.1737C > A (p.F579L), were inherited from their parents, forming a composite heterozygous variation. Genetic testing suggested type I spinal cord atrophy (Figure 3). Thus, based on the clinical manifestations, spinal cord distal muscular atrophy with respiratory distress type 1 was diagnosed.

## Discussion

SMARD1 is a relatively rare autosomal recessive neuronal disease that occurs in the ventral horn of the spinal cord α. The selective loss of motor neurons and subsequent muscle denervation leads to muscle atrophy accompanied by rapid respiratory failure (4). IGHMBP2 mutations result in the expression of pathogenic factors of SMARD1 (5, 6). The IGHMBP2 gene is located at the chromosomal region 11q13.2-q13.4 and is a widely expressed DNA and RNA helicase with multiple cellular functions (7). The majority of known IGHMBP2 mutations are situated in the helicase and R3H domains, leading some scholars to believe that these domains are crucial proteins that trigger severe clinical phenotypes related to ATPase activity. Patients with mild SMARD1 exhibit higher protein expression levels than patients with severe SMARD1. Therefore, the pathogenesis of SMARD1 may be linked to reduced translation or protein degradation (8). Besides SMARD1, IGHMBP2 mutations can also lead to autosomal recessive axonal Charcot–Marie–Tooth disease type 2S (CMT2S), which can cause sensory motor polyneuropathy in childhood, resulting in distal dominant muscle weakness and decreased systemic reflexes without respiratory dysfunction (9). The mutations in SMARD1 are primarily missense mutations located in the helicase domain, while the mutations in CMT2S predominantly consist of nonsense mutations in the 5′ region of the gene, along with truncating, missense, or homozygous frameshift mutations in the last exon. The various mutation combinations in these two diseases result in varying amounts of residual proteins. Cottenie et al. demonstrated that IGHMBP2 protein levels in CMT2S patients were significantly higher than those in SMARD1 patients, indicating that clinical phenotypic differences are related to residual IGHMBP2 protein levels (9).

SMARD1 patients typically present with phrenic nerve palsy between the ages of six weeks and six months, necessitating mechanical ventilation due to respiratory distress. This is manifested as inspiratory hissing, weak crying, or difficulty eating. Secondly, the patient’s distal limb muscles undergo progressive atrophy. The lower limbs are affected earlier than the upper limbs, and the proximal muscles are affected as the disease progresses. The majority of patients die due to respiratory failure or complications associated with mechanical ventilation (8). Due to the similarity of clinical manifestations between SMARD1 and SMA, some scholars believe that SMARD1 can be described as a mutation type of SMA (8, 10); however, it is rarer and easily confused with 5q-SMA type I. Several points distinguish SMARD1 from SMA. First, SMARD1 cause by mutations in the IGHMBP2 gene, which encodes immunoglobulin μ-binding protein 2, leading to progressive spinal motor neuron degeneration. In 95% of patients with SMA, homozygous deletions or smaller mutations are present in exon 7, leading to truncated nonfunctional survival motor neuron (SMN) proteins. This defect can result in muscle atrophy, weakness, and difficulty eating and breathing (11, 12). After completing whole-exome sequencing, no homozygous deletions or small mutations were found in exon 7. Instead, two heterozygous variants were identified in the IGHMBP2 gene were as follows: M1: NM_002180.3:c.407_408del (p.Ala140Glnfs*1); M2: NM_002180.3:c.1737C > A (p.F579L). These findings confirmed the presence of SMARD1. The M1 variant (NM_002180.3:c.407_408del (p.Ala140Glnfs*1)), rated as pathogenic by the American College of Medical Genetics and Genomics (ACMG) guidelines (evidence of PVS1 + PM2), was considered a pathogenic variant in this patient. This variant is predicted to cause frameshift, premature termination of codons, production of truncated proteins, or nonsense-mediated mRNA degradation (13). There are currently no published reports on the correlation between this mutation and SMARD1. However, the literature has reported the detection of M2 mutations (NM_002180.3:c.1737C > A (p.F579L)) in SMARD1 patients (14, 15). According to ACMG guidelines, the M1 variation may be a pathogenic variant, while the M2 variation may be a polymorphism, indicating an unknown clinical significance (16).

Second, in terms of clinical manifestations, SMARD1-related muscle weakness is more severe in the distal end and lower limbs than in the proximal end and upper limbs (1, 4, 17). This is different from typical SMA muscle weakness with severe proximal involvement (18). The electromyographic results indicated neurogenic damage with obvious distal muscles. In the first 3 months, there was progressive poor limb movement, inability to lift both lower limbs or bend the ankle and knee joints, and no movement at the proximal or distal ends of the limbs. Both hands could be lifted off the bed surface, and limb movement was weak, with obvious distal limb movement. The lower and upper limb muscle strengths are levels 2 and 3, respectively; muscle tension is weak, and the deformed spine is an S-shaped bending deformity. This finding is consistent with the observed SMARD1 muscle weakness. Additionally, SMARD1 is characterized by severe diaphragmatic involvement, and children often experience early respiratory distress or even respiratory failure owing to diaphragmatic paralysis. SMA is often characterized by intercostal muscle atrophy and a bell-shaped chest deformity (19). Multiple blood gas analyses of the patient demonstrated hypoxia and carbon dioxide retention, indicating that the respiratory system was involved earlier with autonomic respiratory dysfunction. Chest films revealed elevation of the diaphragm and its critical involvement. The respiratory disorders in this patient occurred after severe hypotonia, which we believe may be related to the significant phenotypic heterogeneity of IGHMBP2 gene mutations. Luan et al. reviewed and analyzed 20 reported cases of nonrespiratory involvement or delayed SMARD1 (15). Some SMARD1 patients may initially exhibit peripheral neuropathy only, and respiratory dysfunction may become evident during follow-up. In some cases, no signs of diaphragmatic paralysis or respiratory distress may appear until 54 months of age. LUAN et al. suggested that the clinical phenotypic differences in SMARD1 may be linked to IGHMBP2 protein levels (9). Therefore, for patients with highly suspected SMA, for whom homozygous deletions in exon 7 or exons 7 and 8 were not detected via gene testing and small mutations were not found via the SMN1 sequencing method, the possibility of non-SMA must be considered. Therefore, IGHMBP2 gene analysis in combination with whole-exome sequencing is recommended. Additionally, the patient should be distinguished from the following diseases: Guillain–Barré syndrome (GBS), myasthenia gravis (MG), and mitochondrial myopathy (PMM), which cause muscle atrophy and/or respiratory failure. Clinical manifestations, gene sequencing, relevant laboratory tests, and systematic neurological examinations play critical roles in distinguishing SMARD1 from the aforementioned diseases.

SMARD1 is a critical progressive congenital neuromuscular disease; however, many issues regarding its origin and genotype–phenotype correlation are unresolved. Joseph S et al. reported two siblings carrying the same mutated gene; however, their clinical manifestations were completely different. The first sibling died of sudden respiratory failure at 6 months, while the second only experienced mild sleep ventilation deficiency at the age of 12 years (20), indicating a heterogeneous phenotype. Therefore, phenotype spectrum analysis to infer prognostic factors will direct scholars to study SMARD1.

The current primary treatment for SMARD1 is respiratory support. While multidisciplinary nursing has played a crucial role in improving the quality of life and extending the lifespan of these patients in recent years, there is still no effective treatment for this devastating disease. In recent years, new treatment methods, such as neurotrophic factor therapy (21), stem cell therapy (22), and gene therapy (23), have shown potential in preventing or mitigating neurodegenerative effects in SMARD1 patients. Ruiz et al. conducted *in vivo* electrophysiological characterization of the neuromuscular degeneration (nmd) mouse model of SMARD1 and assessed the potential therapeutic effect of a monoclonal antibody (Mab2256) targeting tyrosine kinase receptor C (trkC). They reported a brief improvement in muscle strength in mice, although the antibody did not extend the lifespan of nmd mice (21). In a 2014 study, Simone et al. demonstrated that neural stem cells (NSCs) derived from human-induced pluripotent stem cells (iPSCs) have therapeutic potential in the context of SMARD1. They transplanted neural stem cells derived from iPSCs into the spinal cord of nmd mice and differentiated them within the spinal cord, leading to an improvement in their clinical phenotype by protecting their endogenous motor neurons (22). Gene therapy represents another approach to SMARD1 therapy, with the significant advantage of potentially replacing defective genes. Shababi et al. treated nmd mice with AAV9 vectors targeting IGHMBP2 and observed an extension of the lifespan of nmd mice (23). These emerging treatment methods enable preclinical strategies to be quickly translated into human patients, offering hope for improving the bleak prognosis of this devastating disease.

This report presents a case in which SMARD1 was associated with a classic phenotype, aiming to deepen clinicians’ understanding of the disease. Moreover, with the widespread application of a large number of related gene analyses and whole-exome sequencing, the early phenotypic evaluation, early diagnosis, and life-supportive decisions related to SMARD1 will likely increase.

## Data availability statement

The original contributions presented in the study are included in the article/supplementary material, further inquiries can be directed to the corresponding author.

## Ethics statement

Written informed consent was obtained from the individual(s), and minor(s)’ legal guardian/next of kin, for the publication of any potentially identifiable images or data included in this article.

## Author contributions

CZ: Writing – original draft. ZC: Data curation, Writing – original draft. QC: Data curation, Formal analysis, Writing – original draft. XF: Writing – review & editing.
